# Anti-colorectal cancer of *Ardisia gigantifolia* Stapf. and targets prediction via network pharmacology and molecular docking study

**DOI:** 10.1186/s12906-022-03822-8

**Published:** 2023-01-09

**Authors:** Weibo Dai, Jing Yang, Xin Liu, Quanxi Mei, Weijie Peng, Xianjing Hu

**Affiliations:** 1 Pharmacology Laboratory, Zhongshan Hospital, Guangzhou University of Chinese Medicine, 528401 Zhongshan, PR China; 2 Zhongshan Torch Development Zone People’s Hospital, 528401 Zhongshan, PR China; 3 Shenzhen Baoan Authentic TCM Therapy Hospital, 518101 Shenzhen, PR China; 4 Guangdong Provincial Key Laboratory of Research and Development of Natural Drugs, School of Pharmacy, Guangdong Medical University, 523808 Dongguan, PR China

**Keywords:** *Ardisia gigantifolia* Stapf, Chinese herbal medicine, Colorectal cancer, Network pharmacology, Mechanism prediction, Molecular docking, Molecular dynamics simulation

## Abstract

**Background:**

*Ardisia gigantifolia* Stapf. (AGS), a Chinese folk medicine widely grows in the south of China and several studies reported that AGS could inhibit the proliferation of breast cancer, liver cancer, and bladder cancer cell lines. However, little is known about its anti-colorectal cancer (CRC) efficiency.

**Methods:**

In the present study, a combination of MTT assay, network pharmacological analysis, bioinformatics, molecular docking, and molecular dynamics simulation study was used to investigate the active ingredients, and targets of AGS against CRC, as well as the potential mechanism.

**Results:**

MTT assay showed that three kinds of fractions from AGS, including the n-butanol extract (NBAGS), ethyl acetate fraction (EAAGS), and petroleum ether fraction (PEAGS) significantly inhibited the proliferation of CRC cells, with the IC_50_ values of 197.24, 264.85, 15.45 µg/mL on HCT116 cells, and 523.6, 323.59, 150.31 µg/mL on SW620 cells, respectively. Eleven active ingredients, including, 11-O-galloylbergenin, 11-O-protocatechuoylbergenin, 11-O-syringylbergenin, ardisiacrispin B, bergenin, epicatechin-3-gallate, gallic acid, quercetin, stigmasterol, stigmasterol-3-o-β-D-glucopyranoside were identified. A total of 173 targets related to the bioactive components and 21,572 targets related to CRC were picked out through database searching. Based on the crossover targets of AGS and CRC, a protein-protein interaction network was built up by the String database, from which it was concluded that the core targets would be SRC, MAPK1, ESR1, HSP90AA1, MAPK8. Besides, GO analysis showed that the numbers of biological process, cellular component, and molecular function of AGS against CRC were 1079, 44, and 132, respectively, and KEGG pathway enrichment indicated that 96 signaling pathways in all would probably be involved in AGS against CRC, among which MAPK signaling pathway, lipid, and atherosclerosis, proteoglycans in cancer, prostate cancer, adherens junction would probably be the major pathways. The docking study verified that AGS had multiple ingredients and multiple targets against CRC. Molecular dynamics (MD) simulation analysis showed that the binding would be stable via forming hydrogen bonds.

**Conclusion:**

Our study showed that AGS had good anti-CRC potency with the characteristics of multi-ingredients, -targets, and -signaling pathways.

**Supplementary Information:**

The online version contains supplementary material available at 10.1186/s12906-022-03822-8.

## Background

Colorectal cancer (CRC) is the third most commonly diagnosed cancer worldwide which occurs in the colon or rectum [1]. The incidence of CRC dramatically increased in the past few decades because of the changes in human lifestyle, environment, and aged populations [2]. In 2018, the International Agency for Research on Cancer (IARC) reported that about 1.8 million new cases of CRC were diagnosed (approximately 10.2% of total cancer cases) and 860,000 CRC-associated deaths occurred worldwide (approximately 9.2% of all cancer-related deaths) [3]. The new cases and CRC-associated deaths were predicted to increase to 2.2 million and 1.1 million by 2030, respectively [4]. The etiology of CRC is highly complicated and some CRC are genetically predisposed such as familial adenomatous polyposis (FAP), hereditary nonpolyposis colorectal cancer (HNPCC or Lynch syndrome), Peutz-Jeghers syndrome, and so on [5, 6]. Some modifiable risk factors, including smoking, western-style diet addiction, obesity, diabetes, alcohol over-consumption, physical inactivity, antibiotic abuse, and intestinal microbiota disorder, were reported to play an important role in the pathogenesis of CRC [7, 8].

In the clinic, the conventional treatments of CRC mainly include surgery, chemotherapy/radiotherapy, and targeted drug therapy. The CRC drugs currently used are 5-fluorouracil (5-FU), oxaliplatin, and irinotecan, and monoclonal antibodies newly developed including bevacizumab (a monoclonal antibody against VEGF) and cetuximab (a monoclonal antibody against EGFR). However, the 5-year survival rate of CRC patients in the advanced stages remains poor, being 18.5% in the United States and 27.7% in Europe, respectively [3]. Hence, more effective treatments or alternative remedies for CRC are urgently needed. Traditional Chinese herbal medicine (TCM) is a big resource for new drug development due to its long-time usage in the clinic, and numbers of cancer cases proved its good efficiency in neoplasia prevention. A multi-center prospective cohort study suggested that a long-time usage of TCM increased the survival rate of CRC patients at stages II and III [9]. A retrospective cohort study also indicated that TCM could significantly improve disease-free survival, in particular for patients with stage III CRC [10].

*Ardisia gigantifolia* Stapf. (AGS) is a kind of TCM that widely grows in the south of China. It was first recorded in the book of *Sheng-Cao-Yao-Xing-Bei-Yao* in the Qing dynasty and was commonly used for treating rheumatism, the pain of bones and muscles, and traumatic injury [11]. Previous studies showed that AGS mainly contained phenols, quinones, sterols, coumarins, triterpenoids, volatile oils, and flavonoids [12, 13]. AGS had a wide range of biological properties such as anti-inflammation, -oxidation, -thrombosis, and -cancer [14]. Several studies reported that AGS could inhibit the proliferation of MDA-MB-231 cells (breast cancer) [15], Bel-7402 cells (liver cancer) [16], and EJ cells (bladder cancer) [17]. However, there is no study reporting the anti-CRC efficiency of AGS and its underlying mechanism. This study is designed to investigate the anti-proliferation of AGS against CRC and explore the potential molecular mechanism.

**Fig. 1:**
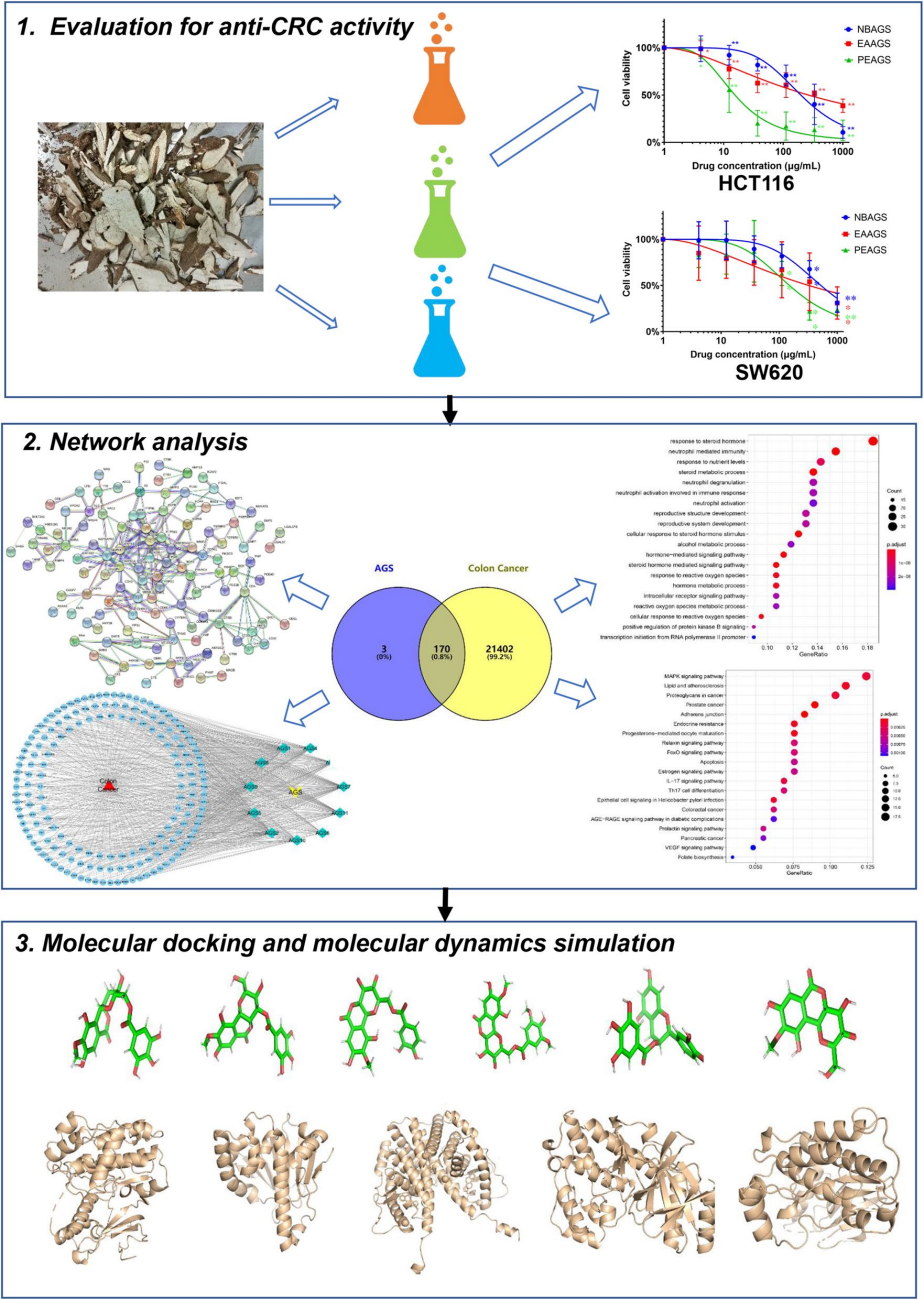
The workflow for the anti-CRC study of *Ardisia gigantifolia* Stapf. (AGS) via a network pharmacological approach

Network pharmacology, an emerging methodology and a useful bioinformatics tool to investigate the complex effects and mechanisms between drugs and diseases at molecular, cellular, tissue, and biologic levels from a systems-level perspective, is now widely used in TCM investigation [18]. In this study, the growth inhibition of AGS against CRC cells was investigated via MTT assay. Network pharmacology was used to analyze the active ingredients, and potential targets of AGS in anti-CRC, as well as predict the possible mechanism and signaling pathway. Furthermore, molecular docking and molecular dynamics simulation were performed to study the binding pattern and stability between active compounds of AGS and therapeutic targets of CRC. The whole work procedure is shown in Fig. 1.

## Materials and methods

### Materials and reagents

The roots and rhizomes of AGS were purchased from Guangzhou Zhixin medicinal herbs Co., Ltd (Lot number: 20,191,201, Guangdong, China) and identified by Professor Suying Tian (Guangdong Pharmaceutical University, Guangzhou, China). The ethanol, petroleum ether (30–60 ℃, 60–90 ℃), ethyl acetate, and n-butanol were obtained from Xilong Chemical Co., Ltd (Guangdong, China). Diatomite was obtained from Dingshengxin Chemical Co., Ltd (Tianjin, China). McCoy’s 5 A medium was purchased from Procell Life Science&Technology Co., Ltd (Wuhan, China). Leibovitz’s L-15 medium was purchased from Dalian Meilun Biotechnology Co., Ltd (Dalian, China). Fetal bovine serum, phosphate-buffered saline, 5% trypsin-EDTA were purchased from Gibco (Carlsbad, CA, United States). Penicillin-streptomycin (P/S) and 3-(4,5-dimethyl-2-thiazolyl)-2,5-diphenyl-2-H-tetrazolium bromide (MTT) were purchased from Beyotime Biotechnology (Shanghai, China).

### Samples preparation of AGS

The roots and rhizomes of AGS were powdered by a high-speed multifunctional crusher (JP-300 A-8, Yongkang, China) to obtain a granulometry of 2 ~ 5 mm. 3 kg of dry powder was combined with 70% ethanol in a round glass flask and extracted by a reflux method for 2 h. This process was repeated three times with the ratio of powder/ethanol of 1:8, 1:6, and 1:4 (w/v), respectively. All the extracted products were collected and dried under a vacuum freeze dryer (LGJ-18, Beijing, China). The extraction yield is 11.28%. Afterward, the ethanolic extracts were dissolved in distilled water and mixed well with diatomite. Dry it in an automatic program-controlled oven with the temperature setting at 70 ℃ and then grind the mixture into a fine powder [19]. The mixture was sequentially macerated and extracted using petroleum ether, ethyl acetate, and n-butanol, respectively, to obtain the three parts of extracts. The extracts were concentrated via a rotary evaporator and lyophilized to obtain three kinds of dry powder, naming PEAGS (, 4.25 mg/g of dried AGS), EAAGS (ethyl acetate extract, 5.1 mg/g of dried AGS), and NBAGS (n-butanol extract, 17.1 mg/g of dried AGS). The extracts were stored at − 35 °C until use.

### Cells culture

Human colorectal cancer cell lines (HCT-116 and SW620) were commercially purchased from the cell resource center of the Shanghai Institutes for Biological Sciences, Chinese Academy of Sciences (Shanghai, China), and cultured in McCoy’s 5 A medium and Leibovitz’s L-15 medium supplemented with 10% fetal bovine serum and 1% penicillin-streptomycin, respectively, at 37 ℃ in a humidified incubator with 5% CO_2_.

### MTT assay

HCT-116 cells (2.5 × 10^3^ / well) and SW620 cells (4.5 × 10^3^ / well) were seeded in 96-well plates and allowed to grow for 24 h, and then treated with different concentrations of PEAGS, EAAGS, NBAGS (0 ~ 1000 µg/mL) for 48 h. After treatment, 20 µL of MTT solution (5 mg/mL) was added to each well and co-incubated for another 4 h at 37 °C. Finally, the supernatant was removed and 150 µL of DMSO was added to each well to dissolve the purple formazan crystals. A microplate reader (PerkinElmer, USA) was used to measure the optical density (OD) of each well at a wavelength of 570 nm. Each experiment was performed in triplicate.

### Network pharmacology analysis

The potential active ingredients in AGS were determined by referring to the published literature by searching 3 databases, the CNKI database (https://www.cnki.net/), Blyun database (http://www.blyun.com/), and Pubmed database (https://pubmed.ncbi.nlm.nih.gov/). The chemical structures of these active ingredients were drawn using the ChemDraw software and were input into the PharmMapper database (http://www.lilab-ecust.cn/pharmmapper/) to obtain the PDB IDs, target names, and fit scores. Then, the potential target set was further summarized according to the fit scores (> 0.7). After excluding the same targets and non-Homo sapiens targets from the screened targets, the PDB IDs were transformed into the gene symbol and gene IDs via the UniProt database (http://www.uniprot.org/). The CRC-related targets were obtained from the GeneCards Human database (https://www.genecards.org) using “colorectal cancer” as a keyword. The crossover genes between AGS and CRC were screened by the R software using the Venn Diagram package after eliminating duplicates. A compound-disease-target network was constructed using the Cytoscape3.7.2 to further explore the therapeutic mechanism of AGS against CRC. The PPI network construction of the predicted targets of AGS in treating CRC was performed based on their interaction data by applying the Interacting Genes/Proteins **(**STRING) database (version 11.0, https://string-db.org/) and visualized using Cytoscape v3.7.2 with the parameters of the minimum required interaction score = 0.9 and hide disconnected nodes in the network. The network can be used to predict the protein interactions (including the physical and functional association between protein targets), among which the nodes represent the intersected target proteins, and edges represent the predicted or validated interactions between proteins. All collective proteins/genes were subjected to Gene Ontology (GO) analysis and Kyoto Encyclopedia of Genes and Genomes (KEGG) enrichment analysis using the database (DAVID, http://david.abcc.ncifcrf.gov/) for annotation, visualization, and integrated discovery. GO analysis consists of Biological Process (BP), Cellular Component (CC), and Molecular Function (MF). KEGG analysis [20–22] aims to identify the significantly altered metabolic pathways of AGS against CRC based on a bioinformatics resource. GO analysis (p < 0.05) and KEGG enrichment analysis (p < 0.05) were visualized using the bioinformatics platform (http://www.bioinformatics.com.cn/).

### Molecular docking analysis

To explore the interactions and binding modes between the active ingredients of AGS and the predicted targets from the network analysis, a molecular docking simulation was carried out using Maestro Schrodinger software 12.5. Six characteristic active components which possess the most targets and the top 5 collective targets were selected for verifying molecular docking. The three-dimensional (3D) sdf format of the 6 active ingredients including 4-O-galloylbergenin, 11-O-galloylbergenin, 11-O-protocatechuoylbergenin, 11-O-syringylbergenin, bergenin, epicatechin-3-gallate was obtained from the PubChem database (https://pubchem.ncbi.nlm.nih.gov/). Structures for the 6 bioactive ligands were prepared via the ‘LigPrep’ module for possible ionization and optical isomers with OPLS3e field forces. The 3D structures of the potential targets including SRC (PBD ID: 3G5D) [23], MAPK1 (PBD ID: 1PME) [24], ESR1 (PBD ID: 1A52) [25], HSP90AA1 (PBD ID: 7lt0) [26], MAPK8 (PBD ID: 1UKI) [27] were downloaded from the RCSB Protein Data Bank (http://www.rcsb.org). All the proteins were processed by undergoing the following procedure before the docking calculations: preprocessing, reviewing, modification, and refinement (including optimization, removing waters, and minimizing the energy using the OPLS3e force field). The binding sites were determined according to the coordinates of the protein ligands. All compounds were docked by docking with standard precision (SP). The docked conformers were evaluated using the docking score. PyMOL 2.4.0 software was used to study the interaction between the docked molecules.

### Molecular dynamic simulation

Molecular dynamics (MD) simulation analysis was performed to evaluate the stability of the protein-small molecule complex in the present study. MD can not only calculate the binding affinities of small molecules within the binding sites in the binding process but also exhibit dynamic conformational changes with the time scale [28, 29]. Therefore, 100 ns of MD simulation was conducted and a total of 10 protein-small molecule complexes were subjected to MD simulations, including SRC-11-O-protocatechuoylbergenin complex, SRC-epicatechin-3-gallate complex, MAPK1-11-O-galloylbergenin complex, MAPK1-epicatechin-3-gallate complex, ESR1-bergenin complex, ESR1-epicatechin-3-gallate complex, HSP90AA1-11-O-protocatechuoylbergenin complex, HSP90AA1-11-O-syringylbergenin complex, MAPK8-11-O-galloylbergenin complex. For the simulation, the AMBER18 package [30] was used to prepare and equilibrate the system with AMBER Force Field ff14SB for the proteins and Force Field GAFF2 for the small molecules [31, 32]. Before the simulation, partial charges for small molecules were calculated by using the antechamber module and Gaussian at the Hartree-Fock (HF) SCF/6-31G* level of theory [33, 34]. A rectangular periodic box of pre-equilibrated three-point transferable intermolecular potential (TIP3P) solvent was used with a minimum distance of 10 Å [35] for solvating the complexes. The electroneutrality of protein/small molecule systems was maintained by adding an appropriate amount of sodium (NA) and chloride (CL) ions if needed. In the next step, the energy minimization (EM) was executed by 2500 steps of steepest descent followed by 2500 steps of a conjugate gradient. After energy minimization, each system was gradually heated from 0 to 298.15 K in a double time of 100 ps with position restraints. After that, the equilibration with position restraint on the protein was performed for 500 ps using NVT (number of particles, volume, and temperature) and NPT (number of particles, pressure, and temperature) ensembles with the temperature of 298.15 K and pressure of 1 bar, respectively. The Particle Mesh Ewald (PME) method was applied to calculate all the long-range electrostatic interactions during the MD simulations process with a radius of 10 Å for coulomb interactions [36]. SHAKE algorithm [37] and Langevin method [38] were performed to constrain all bonds and control the temperature, respectively. Finally, a 100 ns MD was performed with a time step of 2 fs and the MD trajectories were recorded every 10 ps for the following analysis. The root mean square deviation (RMSD), root mean square fluctuation (RMSF), and hydrogen bonding was measured and visually analyzed in the results section.

### Estimation binding free energy via MMPBSA

Molecular mechanics/Poisson-Boltzmann surface area (MM/PBSA) [39–41] was applied to determine thermodynamical stability of small molecules inside the binding sites of the proteins which were computed based on the equations shown below:

1 $$\triangle G_\text{binding}=\triangle G_\text{complex}-\left(\triangle G_\text{receptor}+\triangle G_\text{ligand}\right)=\triangle E_\text{internal}+\triangle E_\text{VDW}+\triangle E_\text{elec}+\triangle G_\text{GB}+\triangle G_\text{SA}$$

In formula (1), the binding free energy ($\varDelta G$_binding_) can be decomposed into five terms: internal energy ($\varDelta E$_internal_), van der Waals ($\varDelta E$_VDW_), electrostatic interaction ($\varDelta E$_elec_), and free energy of solvation which consists of polar ($\varDelta G$_GB_) and non-polar solvation free energy ($\varDelta G$_SA_). Polar solvation free energy was calculated by the GB model (igb = 2) [42] and non-polar solvation free energy was calculated based on the solvent-accessible surface area (SASA) estimated by the LCPO algorithm: $\varDelta G$_SA_ = 0.0072 × $\varDelta$SASA [40]. All the MM/GBSA free energy calculations were performed by using the MMPBSA module in the AMBER 18 package.

### Statistical analysis

The results were expressed as mean ± SEM analyzed with a one-way analysis of variance (ANOVA) using Prism 8.0.1 (GraphPad, San Diego, CA, USA). P < 0.05 was considered to be significantly different.

## Results

### Growth inhibition of AGS on CRC

**Fig. 2:**
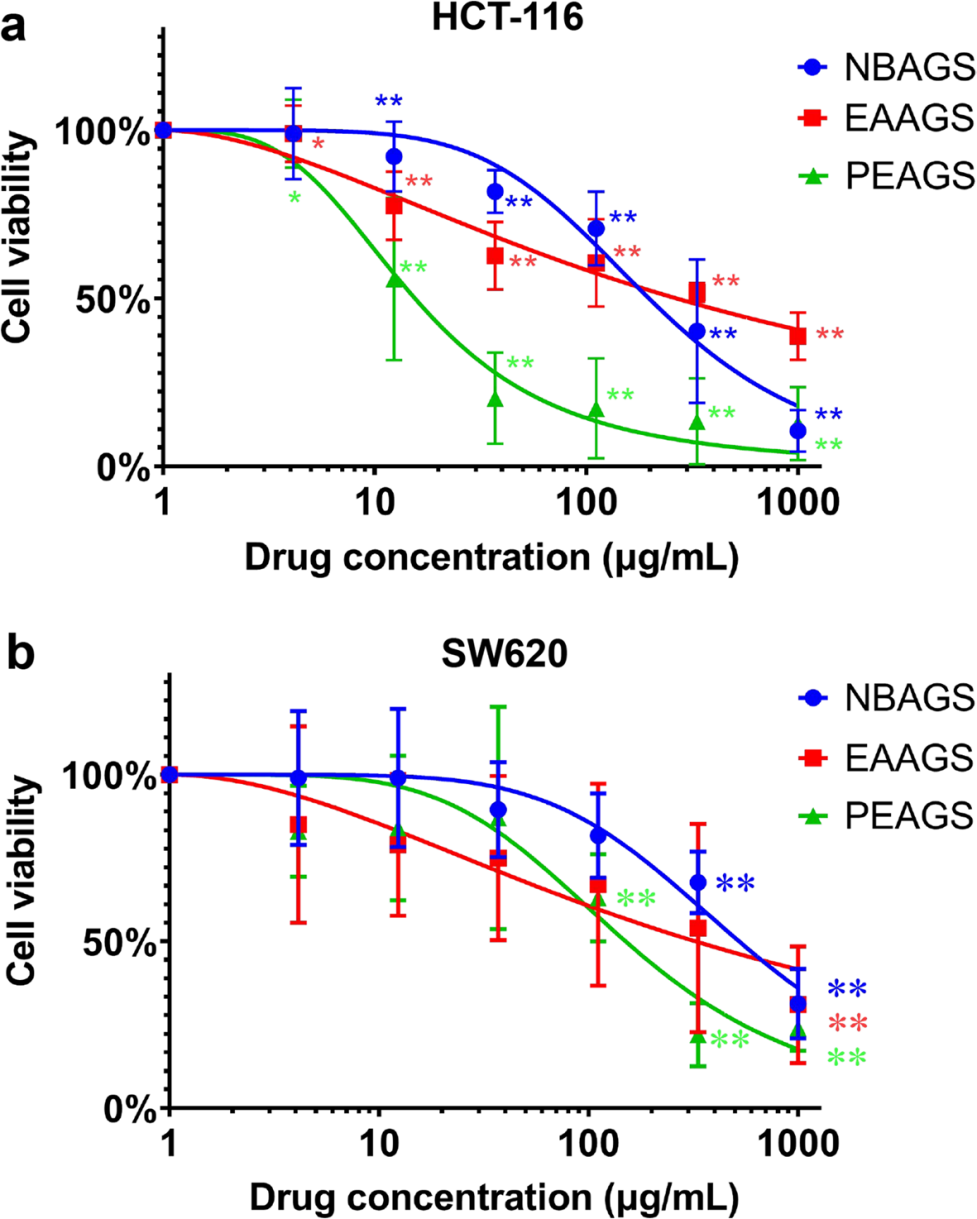
AGS inhibits the cells proliferation of colorectal cancer. HCT-116 and SW620 cell lines were treated with different concentrations of PEAGS, EAAGS, NBAGS (0, 4.1, 12.3, 37.0, 111.1, 333.3, 1000 µg/mL) for 48 h, respectively, and the viability of (**a**) HCT-116 cells, (**b**) SW620 cells were determined by MTT assay. All the experiments were performed in triplicate and data were expressed as mean ± SEM; significance: * *p* < 0.05, ** *p* < 0.01 vs. control

MTT assay was used to evaluate the influence of AGS on the viability of the CRC cells. As shown in Fig. 2, treatments with PEAGS, EAAGS, and NBAGS (0 ~ 1000 µg/mL) for 48 h resulted in the significant inhibition of proliferation on HCT-116 and SW620 cells. The IC_50_ (50% inhibitory concentration) values of NBAGS, EAAGS, and PEAGS were 197.24, 264.85, 15.45 µg/mL on HCT-116 cells (Fig. 2a), and 523.6, 323.59, 150.31 µg/mL on SW620 cells (Fig. 2b), respectively, indicating that the three fractions of AGS could significantly inhibit the growth of CRC cells. These data suggested that AGS would be a good candidate for CRC prevention or therapy.

### Collection of chemical structure and targets information of AGS

**Fig. 3:**
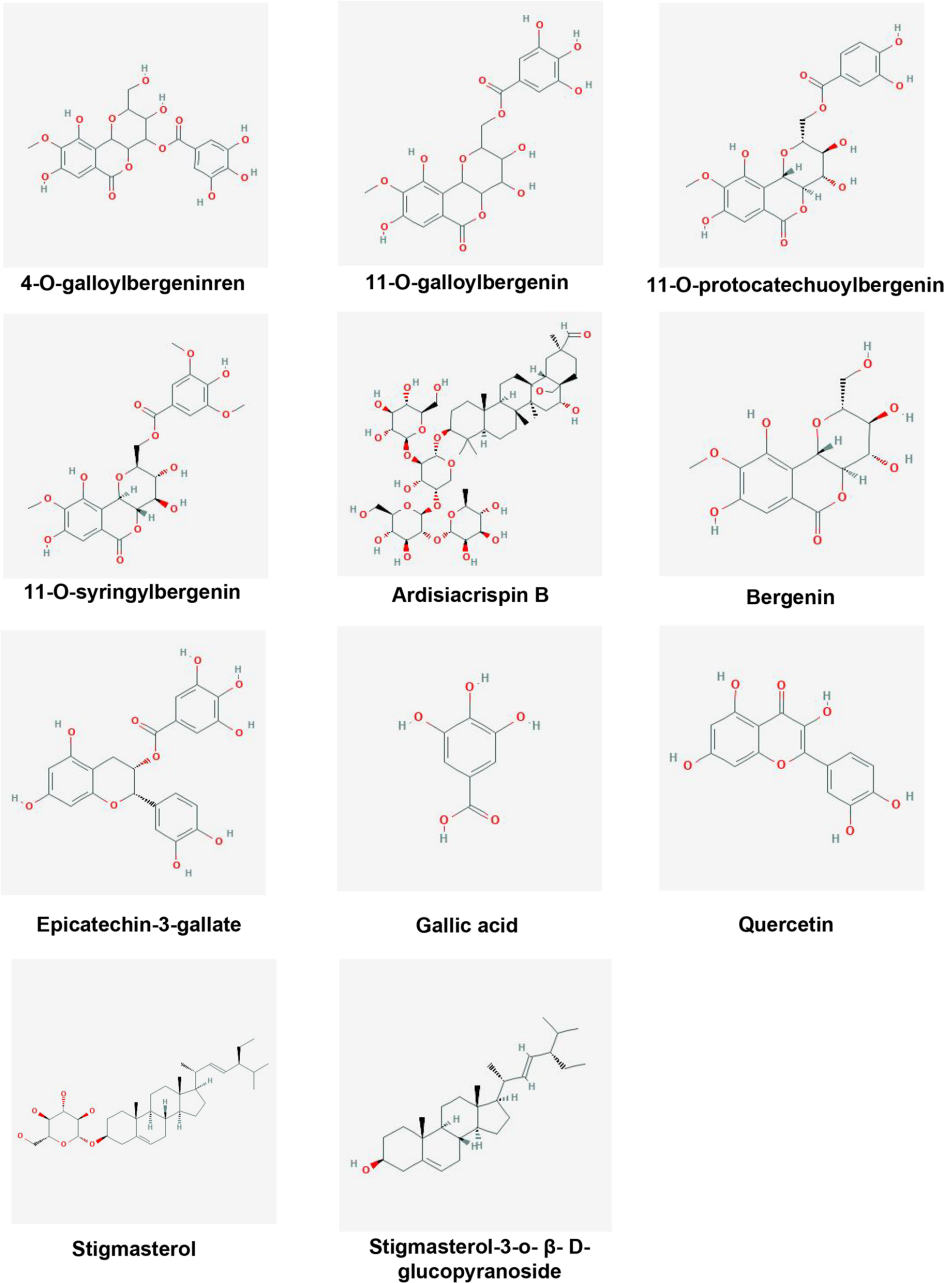
The structures of 11 active compounds from AGS, including 4-O-galloylbergenin, 11-O-galloylbergenin, 11-O-protocatechuoylbergenin, 11-O-syringylbergenin, Ardisiacrispin B, Bergenin, Epicatechin-3-gallate, Gallic acid, Quercetin, Stigmasterol, Stigmasterol-3-o-β-D-glucopyranoside

Network pharmacology analysis was performed to explore the interactions between AGS constituents and potential targets. 11 active ingredients including 4-O-galloylbergenin, 11-O-galloylbergenin, 11-O-protocatechuoylbergenin, 11-O-syringylbergenin, ardisiacrispin B, bergenin, epicatechin-3-gallate, gallic acid, quercetin, stigmasterol, stigmasterol-3-O-β-D-glucopyranoside were identified by database searching (Fig. 3), and the numbers of the targets for 4-O-galloylbergenin, 11-O-galloylbergenin, 11-O-protocatechuoylbergenin, 11-O-syringylbergenin, ardisiacrispin B, bergenin, epicatechin-3-gallate, gallic acid, quercetin, stigmasterol, stigmasterol-3-o-β-D-glucopyranoside were 69, 86, 79, 79, 51, 41, 111, 17, 54, 56, 88, respectively (Supplementary Table S1). A total of 173 targets related to the bioactive components were picked out after deleting the reappeared targets.

### Collection for disease targets

A total of 21,572 targets related to CRC disease were obtained by searching the Genecards database (Supplementary Table S2). The top 10 “high response” genes were screened out according to the score of relevance, including BRCA2, BRCA1, TP53, MSH2, APC, MSH6, MLH1, CDH1, PTEN, and PMS2.

### Prediction for candidate targets of AGS against CRC

**Fig. 4:**
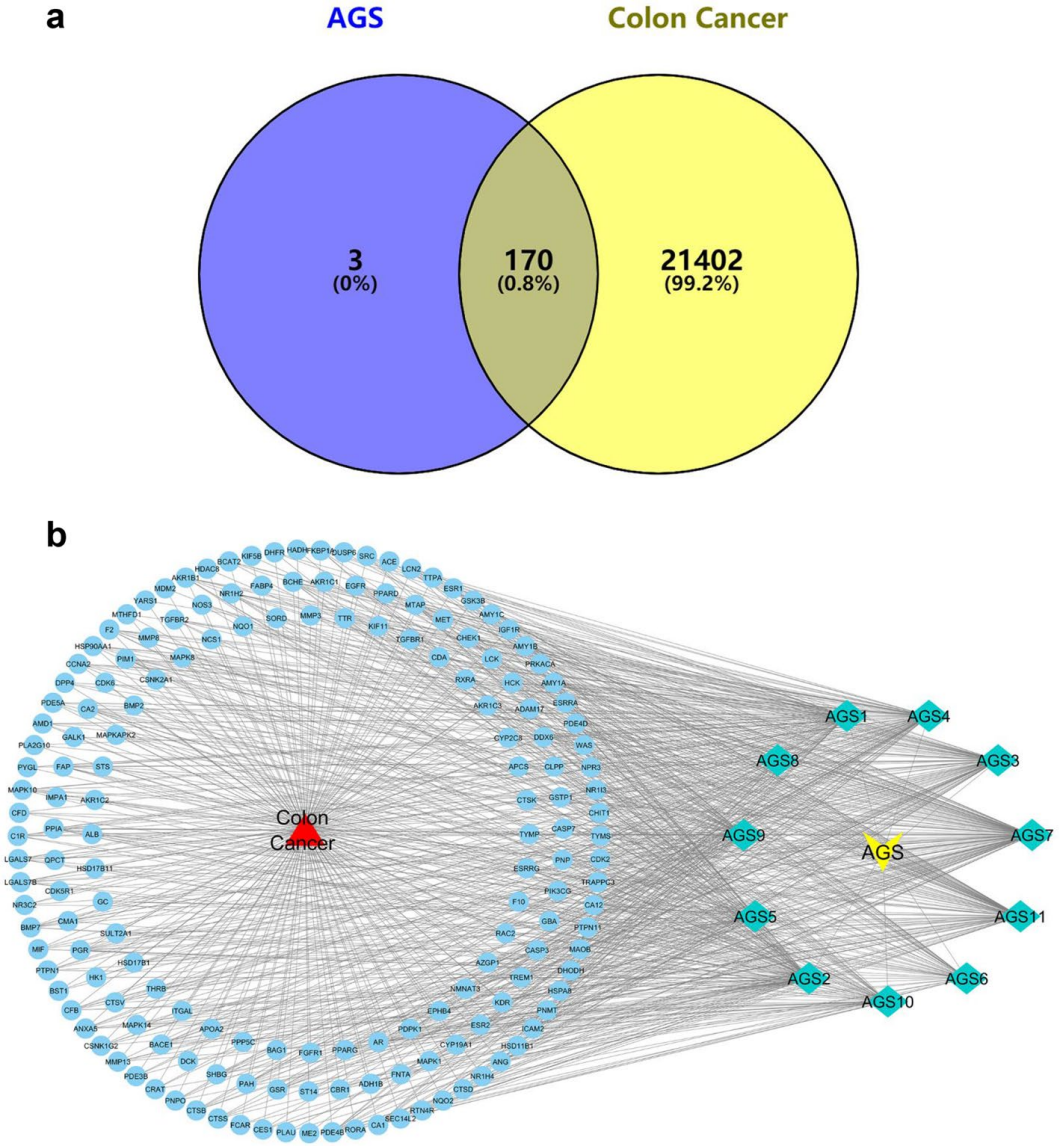
**a** Venn diagram for the predicted targets of AGS against CRC. Eleven active ingredients in AGS may influence the 170 overlapping genes for CRC therapy. **b** Drug-active ingredient-target network diagram. The blue ellipses were representing the 170 overlapping targets and the green quadrilaterals were standing for the 11 active ingredients of AGS

As the Venn diagram showed in Fig. 4a, a total of 170 overlapped genes (Supplementary Table S3) were identified by matching the therapeutic target genes of CRC and target genes of AGS. The “AGS-component-target-CRC” network was built up by importing the crossover genes of AGS & CRC and the potential active components into the system (Fig. 4b).

### PPI network construction and drug-disease key targets prediction

**Fig. 5:**
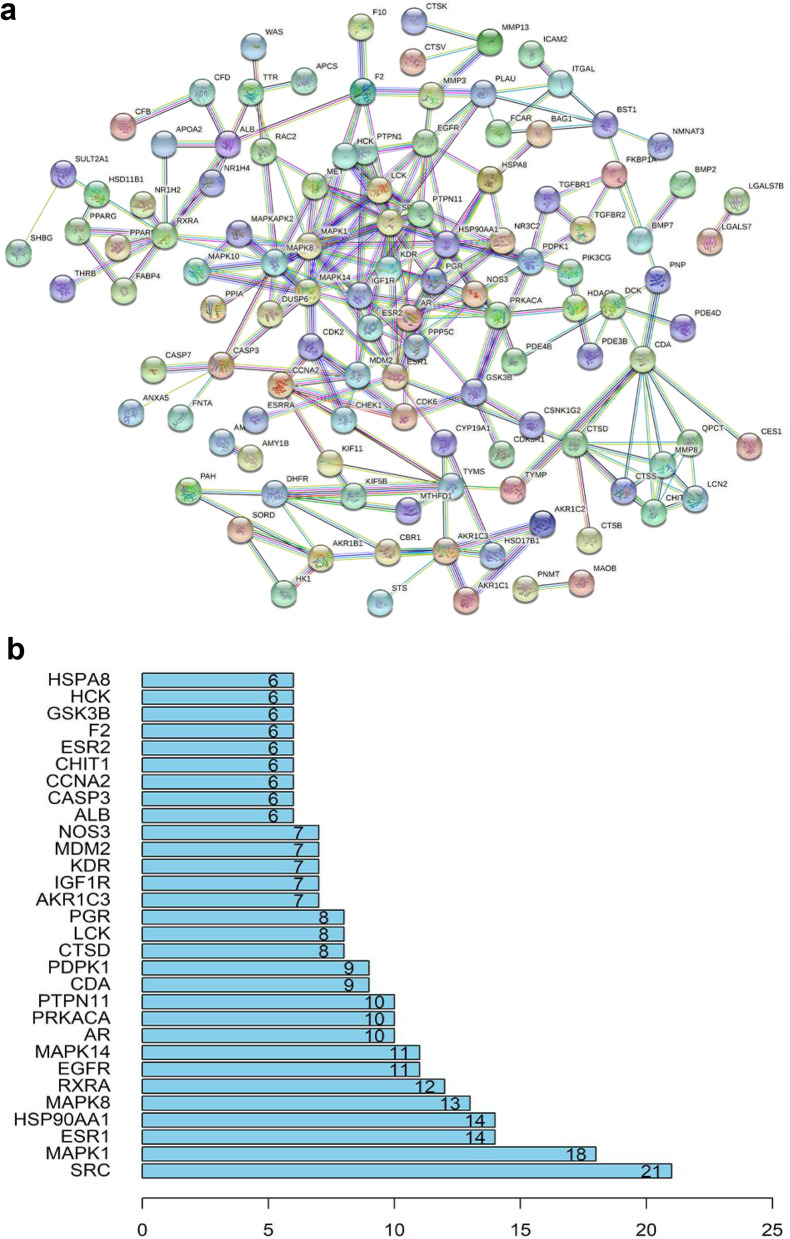
PPI (protein-protein interaction) network. **a** The PPI network was constructed by the String database showing the interactions between the predicted targets of AGS against CRC. The nodes were representing the intersected target proteins, while the edges were indicating the predicted or validated interactions between target proteins. **b** Bar chart for the top 30 targets that had a node degree greater than 6

Based on the crossover targets of AGS & CRC, the PPI network was built up by the String database. As shown in Fig. 5a, there were 169 nodes and 239 edges in the network diagram and the average node degree was 2.83. The top 30 core genes were screened out, of which the node degrees of SRC, MAPK1, ESR1, HSP90AA1, and MAPK8 were greater than 12 (Fig. 5b**)**. According to the results, the core targets were predicted to be SRC, MAPK1, ESR1, HSP90AA1, MAPK8, which had more connections than other genes.

### Enrichment analysis for key targets

**Fig. 6:**
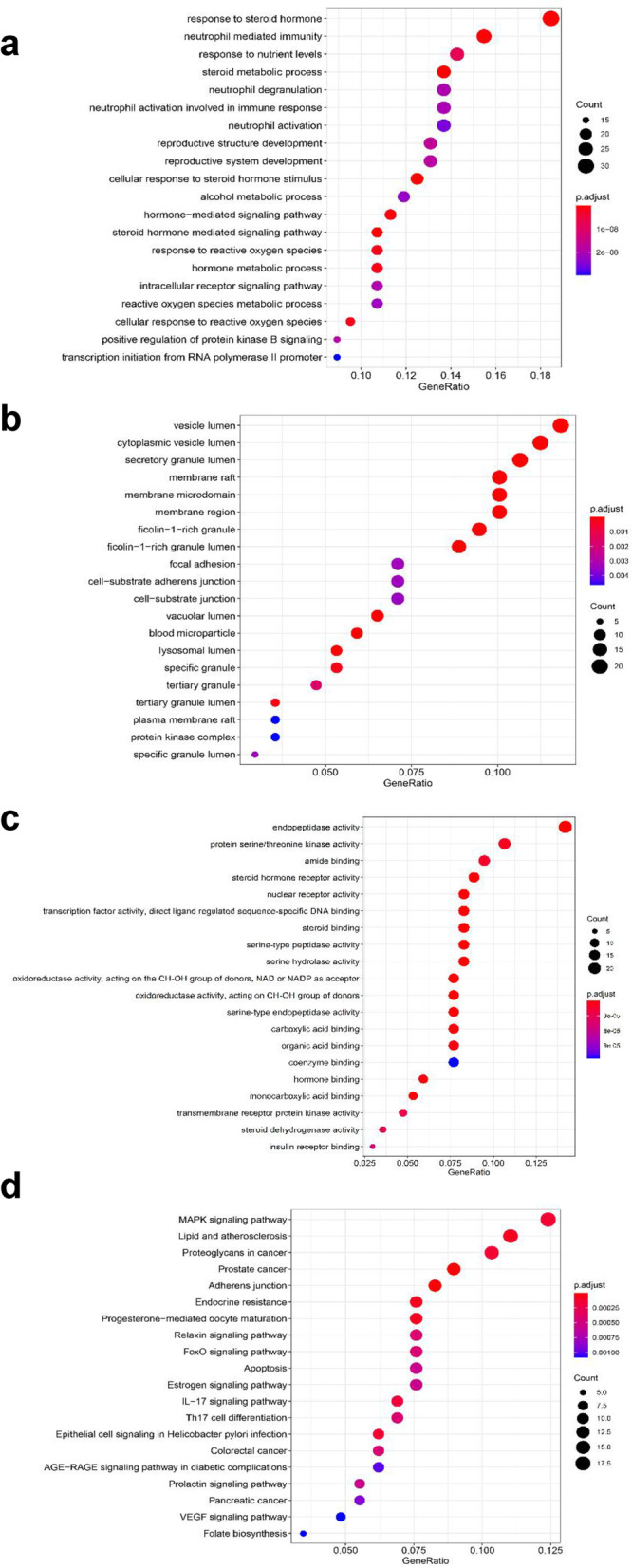
GO and KEGG pathway enrichment analysis. Dot plot showed the GO analysis for AGS in the treatment of CRC, including (**a**) biological process (BP), (**b)** cellular component (CC), (**c**) molecular function (MF). **d** KEGG pathway enrichment analysis for AGS in the treatment of CRC. The dot plot showed the top 20 signaling pathways associated with AGS against CRC

As a result, GO analysis showed that the numbers of BP, CC, and MF of AGS against CRC were 1079, 44, and 132, respectively (Supplementary Table S4), and the top 20 GO analyses of BP, CC, MF had been shown as graphical bubbles. According to the results, AGS would mainly participate in the biological process of steroid metabolic process (Fig. 6a) and the cytoplasm is the major reaction site (Fig. 6b) in the treatment of CRC, during which the central molecular function would probably include the steroid hormone receptor activity, nuclear receptor activity, transcription factor activity, steroid binding and endopeptidase activity (Fig. 6c). Furthermore, a total of 96 signaling pathways (Supplementary Table S5) were screened out through KEGG pathway enrichment analysis, and the top 20 signaling pathways were shown as a bar graph (Fig. 6d), among which the MAPK signaling pathway, lipid, and atherosclerosis, proteoglycans in cancer, prostate cancer, adherens junction, endocrine resistance, progesterone-mediated oocyte maturation, relaxin signaling pathway, foxO signaling pathway, and apoptosis had been proved to be the major pathways related to CRC treatment.

### Molecular docking analysis

**Fig. 7:**
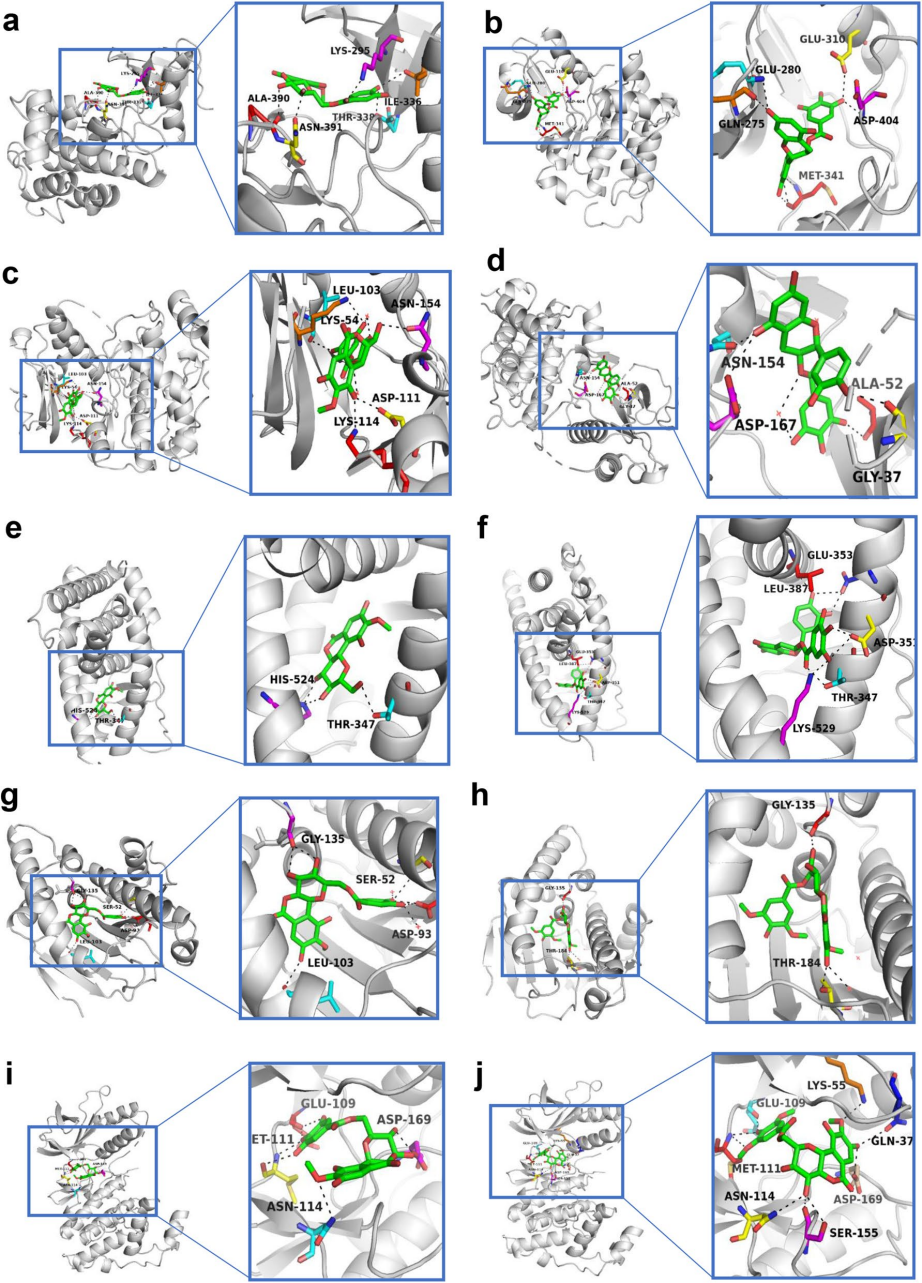
Molecular docking simulation for the compounds of AGS binding the therapeutic targets of colorectal cancer. (**a**) Molecular docking simulation between 11-O-protocatechuoylbergenin and SRC, (**b**) molecular docking simulation between epicatechin-3-gallate and SRC, (**c**) molecular docking simulation between 11-O-galloylbergenin and MAPK1, (**d**) molecular docking simulation between epicatechin-3-gallate and MAPK1, (**e**) molecular docking simulation between bergenin and ESR1, (**f**) molecular docking simulation between epicatechin-3-gallate and ESR1, (**g**) molecular docking simulation between 11-O-protocatechuoylbergenin and HSP90AA1, (**h**) molecular docking simulation between 11-O-syringylbergenin and HSP90AA1, (**i**) molecular docking simulation between 11-O-galloylbergenin and MAPK8, (**j**) molecular docking simulation between 11-O-syringylbergenin and MAPK8

The docking analysis was performed to assess the binding effect and pattern between the active ingredients of AGS and the identified core targets. The results of SP molecular docking between SRC (PBD ID: 3G5D) and the 6 characteristic active components were shown in Table 1. The active sites of SRC were x: 9.27, y: -37.78, z: -4.32, determined by the protein-ligand. Four active compounds, including 11-O-galloylbergenin, 11-O-protocatechuoylbergenin, 11-O-syringylbergenin, epicatechin-3-gallate, can interact with SRC well, with the docking scores ranging from − 7.965 to -6.595. Compared with the SRC ligand (1N1, -6.5), the docking scores of 11-O-protocatechuoylbergenin, epicatechin-3-gallate interacting with SRC were lower, with the docking score of -7.082, -7.965. As shown in Figs. 7a and 11-O-protocatechuoylbergenin could form H bonds with ALA-390 (2.9 Å), ASN-391 (3.1 Å), LYS-295 (3.0 Å), THR-338 (3.3 Å), ILE-336 (3.4 Å), and epicatechin-3-gallate (Fig. 7b) could develop H bonds with GLU-310 (3.1Å), GLU-280 (3.1 Å), ASP-404 (3.1 Å), GLN-275 (3.0 Å), MET-341 (2.7 Å and 2.9 Å). The results of SP molecular docking between MAPK1 (PBD ID: 1PME) and the 6 characteristic active components were shown in Table 1. The active sites of the MAPK1 were x: -12.73, y: 13.48, z: 40.75, determined by the MAPK1 protein-ligand (SB2). All the docking scores of the active compounds were higher than that of the MAPK1 ligand (SB2, -7.509). 11-O-galloylbergenin, epicatechin-3-gallate had relative high-level of interactions with MAPK1, with docking scores of -6.571, -6.43. As shown in Figs. 7c and 11-O-galloylbergenin could develop H bonds with ASN-154(2.8 Å), LEU-103(3.1 Å), LYS-54(3.3 Å), ASP-111(2.8 Å), LYS-114(3.0 Å), and epicatechin-3-gallate (Fig. 7d) developed H bonds with ASN-154(2.6 Å), ALA-52(3.4 Å), ASP-167(3.4 Å), GLY-37(2.8 Å). The SP molecular docking results of ESR1 (PBD ID: 1A52) with the 6 characteristic active components were shown in Table 1. The active sites of the ESR1 were x: 95.12, y: 92.31, z: 109.75, determined by the ESR1 protein-ligand. The 4-O-galloylbergenin failed to dock with ESR1. According to the docking scores results, 11-O-galloylbergenin, 11-O-protocatechuoylbergenin, 11-O-syringylbergenin could not interact with ESR1 well, with the scores ranging from − 5.91 to -1.7, while bergenin, epicatechin-3-gallate had greater levels of interactions with ESR1, with the docking scores of -8.133, -8.797, lower than the docking score of the ligand (EST, -6.7). As shown in Fig. 7e, bergenin could develop H bonds with HIS-524 (2.7 Å and 2.8 Å) and THR-347 (3.0 Å), and epicatechin-3-gallate (Fig. 7f) could form H bonds with GLU-353 (4.9 Å and 2.5 Å), LEU-387 (3.3 Å), ASP-351 (2.6 Å and 3.2 Å), THR-347 (2.7 Å), LYS-529 (2.8 Å). The SP molecular docking results of HSP90AA1 (PBD ID: 7lt0) with the 6 characteristic active components were shown in Table 1. The active sites of the HSP90AA1 were x: -31.94, y: -10.74, z: -25.24, determined by the HSP90AA1 protein-ligand. According to the docking scores, all 6 active compounds didn’t interact with HSP90AA1 well, with the scores ranging from − 8.847 to -6.955, higher than the docking score of the ligand (ONJ, -9.144). 11-O-protocatechuoylbergenin, 11-O-syringylbergenin had relatively high levels of interactions with HSP90AA1, with docking scores of -8.847, -8.288. As shown in Fig. 7 g, 11-O-protocatechuoylbergenin could develop H bonds with GLY-135 (2.8 Å and 2.8 Å), SER-52 (3.1 Å), ASP-93 (2.9 Å), LEU-103 (3.2 Å), and 11-O-syringylbergenin (Fig. 7 h) could form H bonds with GLY-135 (2.7 Å), THR-184 (3.1Å). The SP molecular docking results of MAPK8 (PBD ID: 1UKI) with the 6 characteristic active components were shown in Table 1. The active sites of MAPK8 were x: 2.22, y: 39.06, z: 29.48, determined by the protein-ligand. All the active compounds could not interact with MAPK8 well, with the scores ranging from − 7.374 to -5.835, higher than the docking score of the MAPK8 ligand (537, -9.397). 11-O-galloylbergenin, 11-O-syringylbergenin had high levels of interactions with MAPK8, with docking scores of -7.106, -7.374. As shown in Figs. 7i and 11-O-galloylbergenin could develop H bonds with GLU-109 (3.0 Å and 3.5 Å), ASP-169 (2.7 Å and 2.8 Å), MET-111 (3.4 Å and 3.0 Å), ASN-114 (3.5 Å), and 11-O-syringylbergenin (Fig. 7j) could develop H bonds with LYS-55(3.3 Å), GLN-37 (3.5 Å), GLU-109 (3.0 Å), MET-111 (3.3 Å), ASN-114 (3.4 Å and 3.0 Å), SER-155 (3.0 Å and 2.9 Å), ASP-169 (2.8 Å).

**Table 1:** Interactions between the major active compounds from AGS and CRC therapeutic targets

Targets	Compounds	Docking score	Glide score	Glide emodel
SRC	1N1	-6.503	-7.13	-65.679
	4-O-galloylbergenin	-5.875	-5.968	-66.129
	11-O-galloylbergenin	-6.595	-6.673	-78.65
	11-O-protocatechuoylbergenin	-7.082	-7.127	-77.558
	11-O-syringylbergenin	-7.071	-7.113	-76.722
	Bergenin	-6.136	-6.159	-55.714
	Epicatechin-3-gallate	-7.965	-8.037	-81.097
MAPK1	SB2	-7.509	-7.509	-46.449
	4-O-galloylbergenin	-5.71	-5.803	-59.556
	11-O-galloylbergenin	-6.571	-6.648	-69.45
	11-O-protocatechuoylbergenin	-5.741	-5.786	-63.71
	11-O-syringylbergenin	-5.473	-5.515	-63.202
	Bergenin	-4.264	-4.288	-41.594
	Epicatechin-3-gallate	-6.43	-6.503	-63.488
ESR1	EST	-6.76	-6.76	-35.044
	4-O-galloylbergenin	/	/	/
	11-O-galloylbergenin	-5.91	-5.987	-33.049
	11-O-protocatechuoylbergenin	-1.7	-1.8	-26.463
	11-O-syringylbergenin	-2.659	-2.702	-16.573
	Bergenin	-8.133	-8.157	-42.469
	Epicatechin-3-gallate	-8.797	-8.87	-32.918
HSP90AA1	ONJ	-9.144	-9.936	-78.187
	4-O-galloylbergenin	-7.791	-7.883	-78.109
	11-O-galloylbergenin	-7.531	-7.608	-82.335
	11-O-protocatechuoylbergenin	-8.847	-8.892	-83.51
	11-O-syringylbergenin	-8.288	-8.331	-89.663
	Bergenin	-6.955	-6.979	-55.288
	Epicatechin-3-gallate	-7.908	-7.98	-79.796
MAPK8	537	-9.397	-9.397	-54.437
	4-O-galloylbergenin	-5.902	-5.995	-55.432
	11-O-galloylbergenin	-7.106	-7.183	-75.825
	11-O-protocatechuoylbergenin	-5.835	-5.88	-69.133
	11-O-syringylbergenin	-7.374	-7.416	-80.646
	Bergenin	-6.417	-6.44	-54.745
	Epicatechin-3-gallate	-7.157	-7.23	-74.634

Altogether, the results showed that the target proteins SRC and ESR1 had stronger docking capability with AGS than the other targets. Four compounds (11-O-galloylbergenin, 11-O-protocatechuoylbergenin, 11-O-syringylbergenin, epicatechin-3-gallate) had a lower docking score than the ligand-protein of SRC, and two compounds (bergenin, epicatechin-3-gallate) docked better than the ligand-protein of ESR1 (Table 1**)**, which indicated that AGS had multiple ingredients and multiple targets against CRC.

### Stability analysis

**Fig. 8:**
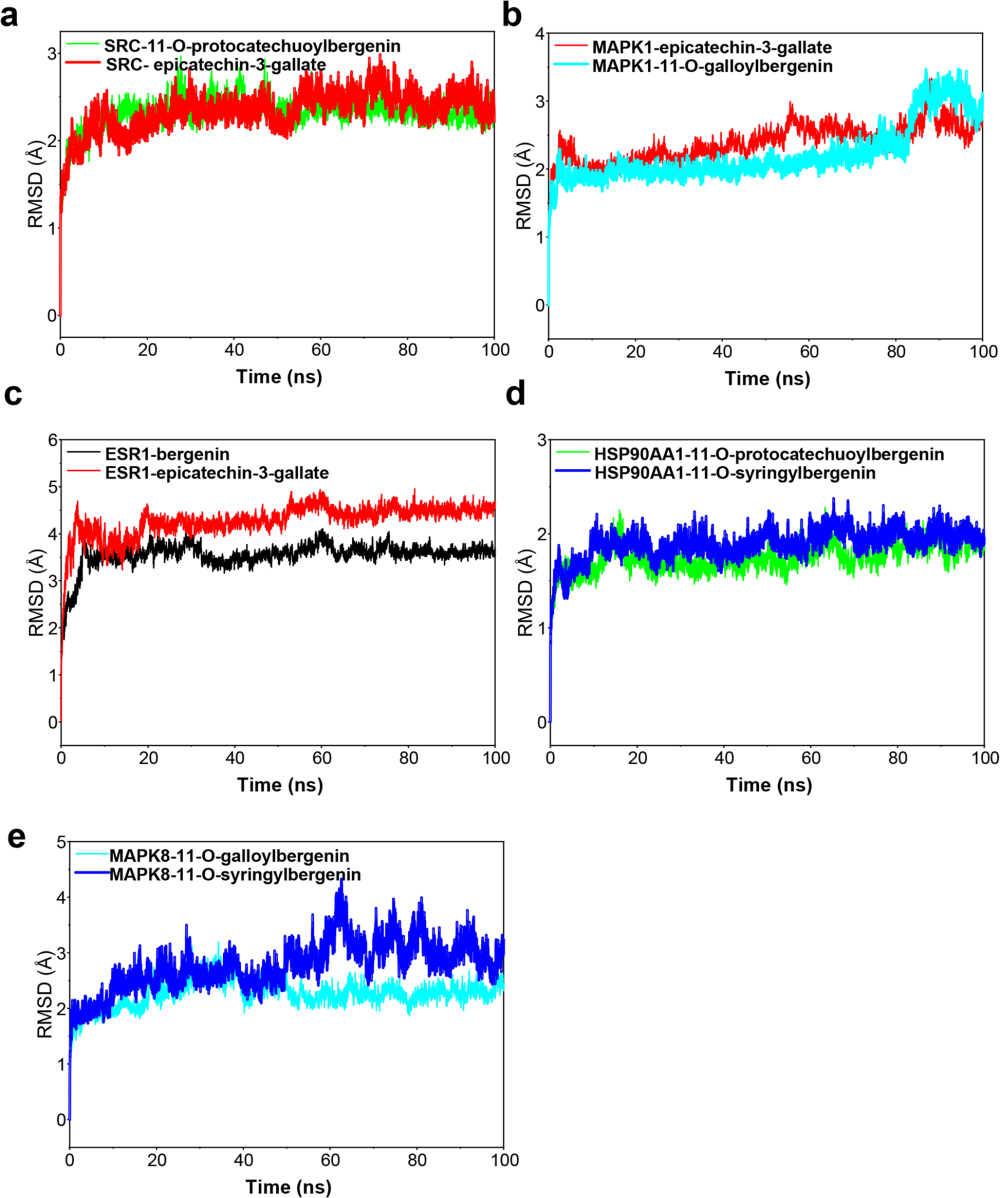
The evolution of RMSD for 10 complexes with time in molecular dynamics simulation. RMSD data for (**a**) SRC-11-O-protocatechuoylbergenin complex and SRC-epicatechin-3-gallate complex, (**b**) MAPK1-epicatechin-3-gallate complex and MAPK1-11-O-galloylbergenin complex, **(c)** ESR1-bergenin complex and ESR1-epicatechin-3-gallate complex, (**d**) HSP90AA1-11-O-protocatechuoylbergenin complex and HSP90AA1-11-O-syringylbergenin complex, (**e**) MAPK8-11-O-galloylbergenin complex and MAPK8-11-O-syringylbergenin complex

In this study, molecular dynamics (MD) simulation was used to analyze the stability of compounds from AGS binding multiple targets including SRC, MAPK1, ESR1, HSP90AA1, and MAPK8. We analyzed the root mean square deviation (RMSD) plot, presenting the stability of protein, and the higher of RMSD value is, the more violent the fluctuation is. On the contrary, the protein binding is stable. As shown in Fig. 8, all the systems were generated and submitted for 100 ns in MD simulations. Among all the systems, HSP90AA1-11-O-protocatechuoylbergenin complex and HSP90AA1-11-O-syringylbergenin complex (Fig. 8d**)** were the most stable systems, with the lowest RMSD value fluctuating stably within the range of 2 Å, which were followed by SRC-11-O-protocatechuoylbergenin complex, SRC-epicatechin-3-gallate complex (Fig. 8a) and MAPK8-11-O-syringylbergenin complex (Fig. 8e), fluctuating steadily at about 2.4 Å. The RMSD values of ESR1-bergenin complex and ESR1-epicatechin-3-gallate complex (Fig. 8c) were much higher than other systems, while these two systems had a high degree of convergence and stable fluctuations in the late simulation week, suggesting that they were also very stable in the middle and late simulation. For MAPK1-epicatechin-3-gallate complex, MAPK1-11-O-galloylbergenin complex **(**Fig. 8b**)**, and MAPK8-11-O-galloylbergenin complex (Fig. 8d), the fluctuations were slightly strong during the simulation process, while the RMSD did not exceed the 0.3 Å threshold [41], indicating that there were no remarkable conformational changes.

### Residue mobility analysis

**Fig. 9:**
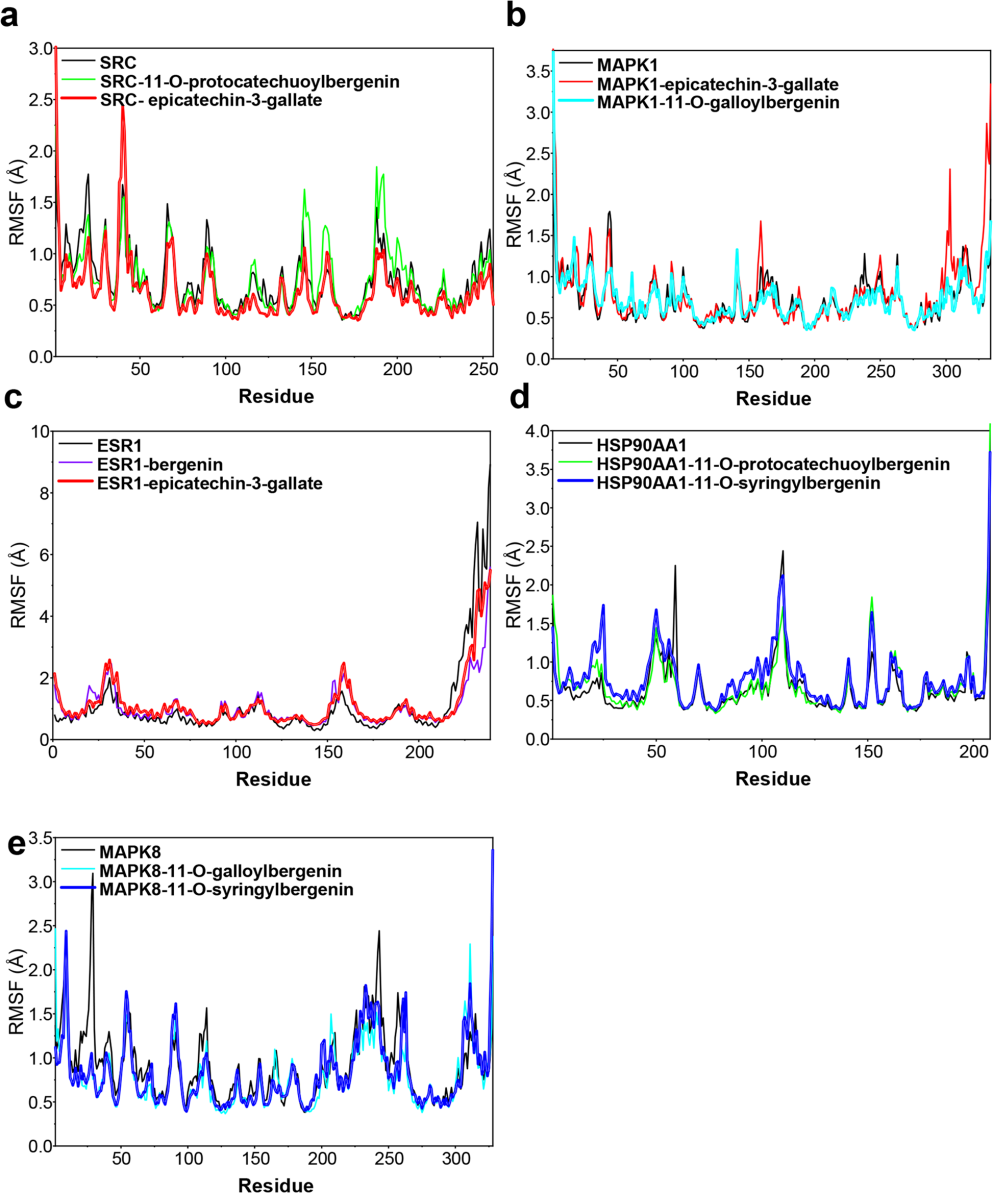
The RMSF values for 10 complexes in the molecular dynamics simulation, including (**a**) SRC-11-O-protocatechuoylbergenin complex and SRC-epicatechin-3-gallate complex, (**b**) MAPK1-epicatechin-3-gallate complex and MAPK1-11-O-galloylbergenin complex, (**c**) ESR1-bergenin complex and ESR1-epicatechin-3-gallate complex, (**d**) HSP90AA1-11-O-protocatechuoylbergenin complex and HSP90AA1-11-O-syringylbergenin complex, (**e**) MAPK8-11-O-galloylbergenin complex and MAPK8-11-O-syringylbergenin complex

To examine the structural-flexibility effect of small molecules on target proteins in the process of molecular dynamics simulation. We analyzed the root mean square fluctuation (RMSF) plot of the 10 complexes. In general, the flexibility of the target protein decreases after a compound binding to the protein, to stabilize and activate the protein [43]. As shown in Fig. 9, the RMSF values (color line) of ESR1-bergenin complex, ESR1-epicatechin-3-gallate complex, HSP90AA1-11-O-protocatechuoylbergenin complex, HSP90AA1-11-O-syringylbergenin complex, MAPK8-11-O-galloylbergenin complex, and MAPK8-11-O-syringylbergenin complex were lower than these in the apo form of corresponding proteins (black line) at multiple amino acid sequences (such as ESR1 210–250, HSP90AA1 55–60, 100–110, MAPK8 20–45, 60–70,100–115 regions). These results indicated that the fluctuations of backbone atoms in these proteins were significantly reduced after binding small molecules, and the proteins became more stable compared with apo-form protein. However, the same changes were not observed in the complexes of SRC and MAPK1, meaning that the fluctuation increased due to the binding of small molecules.

### Hydrogen bonds analysis

**Fig. 10:**
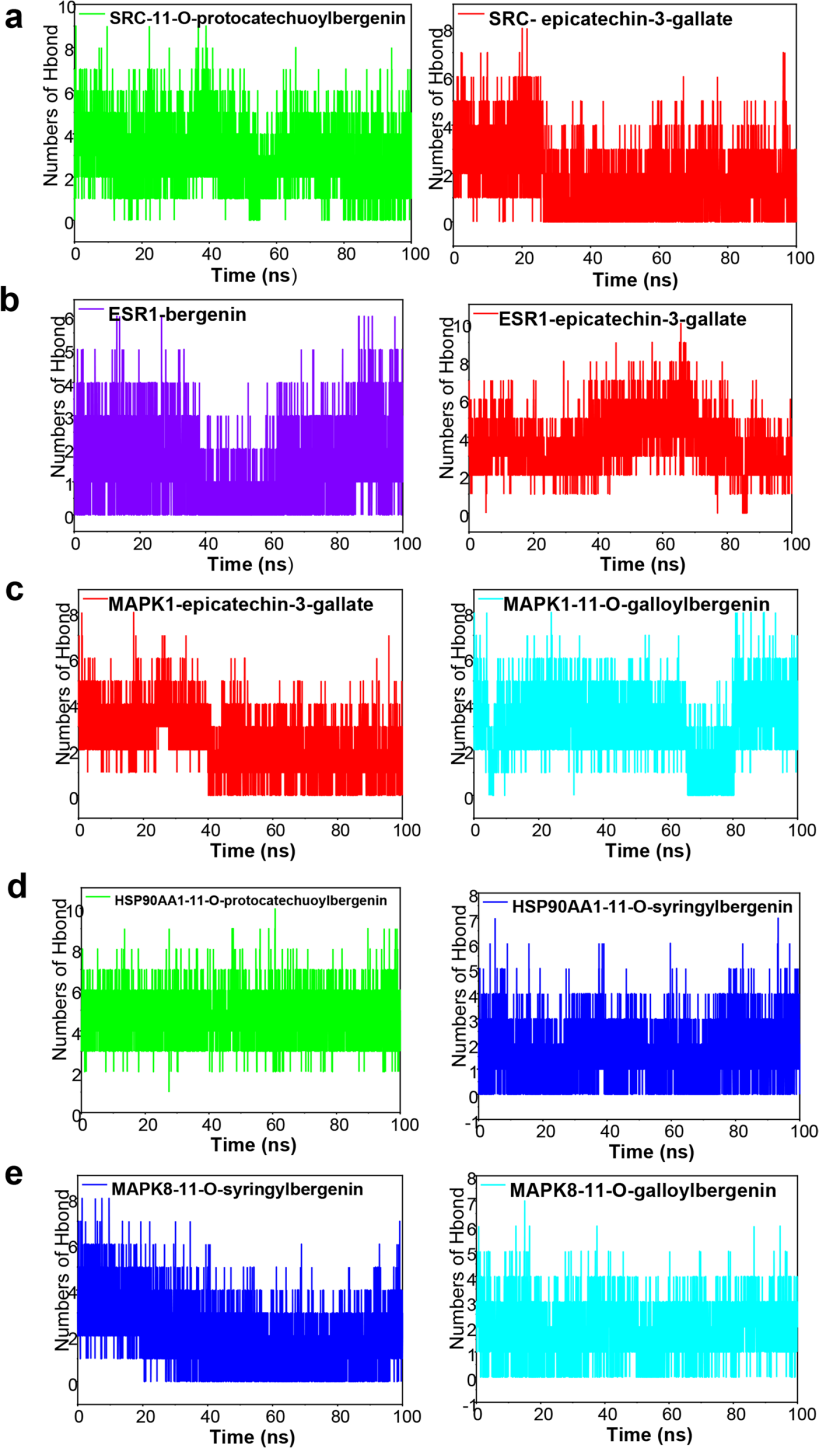
The comparative hydrogen bonding for the 10 complexes in 100 ns. Hydrogen bonding data for (**a**) SRC-11-O-protocatechuoylbergenin complex and SRC-epicatechin-3-gallate complex, (**b**) MAPK1-epicatechin-3-gallate complex and MAPK1-11-O-galloylbergenin complex, (**c**) ESR1-bergenin complex and ESR1-epicatechin-3-gallate complex, (**d**) HSP90AA1-11-O-protocatechuoylbergenin complex and HSP90AA1-11-O-syringylbergenin complex, (**e**) MAPK8-11-O-galloylbergenin complex and MAPK8-11-O-syringylbergenin complex

The hydrogen bond is one of the strongest non-covalent interactions between small molecule compounds and proteins. During the molecular dynamic simulation, we analyzed the number of hydrogen bonds in all 100 ns of 10 complexes. As shown in Fig. 10, the hydrogen bonds could be seen in all complexes and the average number of hydrogen bonds were 4 in ESR1-epicatechin-3-gallate complex, HSP90AA1-11-O-protocatechuoylbergenin complex, MAPK1-11-O-galloylbergenin complex, SRC-11-O-protocatechuoylbergenin complex was 4, being much more than other complexes, which suggested that these four complexes were more stable than others. The ESR1-bergenin complex, HSP90AA1-11-O-syringylbergenin complex, MAPK1-epicatechin-3-gallate complex, MAPK8-11-O-galloylbergenin complex, MAPK8-11-O-syringylbergenin complex and SRC-epicatechin-3-gallate complex formed less than 4 hydrogen bonds in the middle and late stages of simulation, indicating that the formation of these complexes may not depend on hydrogen bonds.

### MMPBSA and binding free energy analysis

In this study, MD trajectories were fully sampled to accurately calculate the binding energy between small molecules and proteins based on MM/GBSA calculation method. As shown in Table 2, the binding energy of SRC-11-O-protocatechuoylbergenin complex, SRC-epicatechin-3-gallate complex, MAPK1-11-O-galloylbergenin complex, MAPK1-epicatechin-3-gallate complex, ESR1-bergenin complex, ESR1-epicatechin-3-gallate complex, HSP90AA1-11-O-protocatechuoylbergenin complex, HSP90AA1-11-O-syringylbergenin complex, MAPK8-11-O-galloylbergenin complex, MAPK8-11-O-syringylbergenin complex were − 27.4629, -24.6023, -26.5586, -17.3903, -29.1308, -33.3846, -44.7534, -33.9108, -26.3971 and − 35.3019 kcal/mol, respectively. The binding energy values of all complexes were below − 15 kcal/mol, indicating that they interacted stably. Especially, for the complexes of ESR1-epicatechin-3-gallate, HSP90AA1-11-O-protocatechuoylbergenin, HSP90AA1-11-O-syringylbergenin, and MAPK8-11-O-syringylbergenin, the binding energy was lower than − 30 kcal/mol, suggesting the strong binding capabilities between the small molecules and target proteins.

**Table 2:** Binding free energies and energy components predicted by MM/GBSA (kcal/mol)

Targets	Compounds	ΔE_vdW_	ΔE_elec_	ΔG_GB_	ΔG_SA_	ΔG_bind_
SRC	11-O-protocatechuoylbergenin	-43.898	-9.949	32.4601	-6.0759	-27.4629
	epicatechin-3-gallate	-38.5333	-19.4689	39.3057	-5.9109	-24.6023
MAPK1	11-O-galloylbergenin	-32.4351	-57.4738	69.5228	-6.1725	-26.5586
	epicatechin-3-gallate	-36.0848	-34.9626	59.5845	-5.9323	-17.3903
ESR1	epicatechin-3-gallate	-40.1026	86.9746	-73.2195	-7.0408	-33.3846
	bergenin	-37.8416	-28.1287	42.872	-6.0325	-29.1308
HSP90AA1	11-O-protocatechuoylbergenin	-43.6791	-62.712	68.853	-7.2154	-44.7534
	11-O-syringylbergenin	-50.4167	-16.1588	39.7165	-7.0517	-33.9108
MAPK8	11-O-galloylbergenin	-36.3445	-73.5254	89.2117	-5.7389	-26.3971
	11-O-syringylbergenin	-48.1223	-35.6195	55.4045	-6.9646	-35.3019

## Discussion

Colorectal cancer ranks third among the causes of cancer-related death and carries a huge global health burden [44]. Although the development of diagnostic and therapeutic technology has improved the survival of patients, approximately 0.8 million CRC-related deaths occur worldwide each year [3]. Chemotherapeutic agents such as ramucirumab, aflibercept, and bevacizumab have been developed to use in the clinic, while they would induce non-negligible side effects. Surgery remains the preferred treatment and 5FU is still the most commonly used drug for CRC therapy [45]. Hence, new agents with more efficacy and low side effects are urgently needed for CRC therapy.

Traditional Chinese herbal medicine (TCM) has been used for thousands of years in China, with the characteristics of definite therapeutic effects and low side effects, and has been used as an alternative therapy for cancer patients. Shi et al. found that TCM could significantly improve the disease-free survival of cancer patients, in particular with stage III patients [10], and Xu et al. found that long usage of TCM herbals could improve the survival outcomes in stages II and III CRC patients in China [9]. Meanwhile, a series of compounds derived from TCM such as baicalein (a constituent of *Scutellaria baicalensis* Georgi, Huangqin), curcumin (a constituent of *Curcuma longa* L., Jianghuang), berberine (a constituent of *Coptis chinensis* Franch., Huanglian) have been demonstrated to have good efficiencies in cancer treatment [46–48]. Therefore, TCM can be recognized as an important resource for antitumor new drug development.

In our study, we verified that the 3 parts of AGS extracts, PEAGS, EAAGS, and NBAGS showed a good anti-CRC effect against HCT-116 and SW620 cells proliferation determined by MTT assay. Literature searching indicated that the flavones (4-O-galloylbergenin, 11-O-galloylbergenin, 11-O-protocatechuoylbergenin, 11-O-syringylbergenin, bergenin, epicatechin-3-gallate, quercetin) and terpenoids (ardisiacrispin B, gallic acid, stigmasterol, stigmasterol-3-o-β-D-glucopyranoside) were the major pharmacological active ingredients of AGS [49, 50]. Chang found that a high intake of flavones (such as quercetin) may reduce the risk of colon cancer [51]. Bergenin, one of the active ingredients of AGS, had been found to inhibit bladder cancer progression by activating the PPARγ/PTEN/Akt signal pathway [52]. Epicatechin-3-gallate was also found to possess a series of pharmacological and physiological properties, including induction of phase II enzymes, mediation of anti-inflammation response, regulation of cell proliferation and apoptosis effects, and prevention of tumor angiogenesis, invasion, and metastasis [53]. Numbers of studies indicated that quercetin had antitumor effects by promoting cell apoptosis, and autophagy, and inhibiting MAPK/Erk, PI3K/Akt, and NF-κB signaling pathways [54, 55].

Network pharmacology, an emerging discipline that is commonly used to predict the potential targets and mechanisms based on the accumulation of evidence from big data, would provide strong support and accelerate the process of new drug development. In our study, we utilized the network pharmacological methodology to explore the pharmacological mechanism of the active components of AGS against CRC, and molecular docking and molecular dynamic simulation were used to investigate the docking pattern and capability between major active compounds of AGS and potential target proteins. The results showed that 11 active ingredients in AGS would have effects on 170 overlapping genes that acting important roles in CRC treatment. PPI network study showed that SRC, MAPK1, ESR1, and HSP90AA1 were the most correlated proteins, followed by MAPK8. SRC family kinase is a key mediator of cellular tumor-promotion genic signals linked with tumor proliferation, migration, and invasion, and SRC could be activated in CRC through various mechanisms, including the regulations of the SRC-STAT3 signaling pathway, SRC-CTNNB1, and macro-autophagy/autophagy pathways [56, 57]. Therefore, SRC inhibitors have been considered ideal therapeutic agents. Mitogen-activated protein kinase (MAPK) is a kinase family that converts extracellular stimuli into various cellular responses and participates in the process of disease occurrence and development [58]. Multiple MAPK pathways are associated with the processes of mitosis, differentiation, metabolism, motility, apoptosis, and survival of eukaryotic cells [59]. MAPK1 (mitogen-activated protein kinase 1), an important member of the MAPK kinase family, includes a highly conserved serine/threonine kinase domain [60], and has been reported to be a target for microRNAs, such miR-212 [61], miR454 [62] and miR-422a [63]. MAPK8 at phosphorylation status was also found to be associated with germ cell apoptosis and redistribution of the Bcl2-modifying factor [64]. ESR1, one of the estrogen receptors (ER), is activated by the sex hormone estrogen. A study showed that approximately 5% of primary tumor patients harbored ESR1 mutation which was increased to 30% ~ 40% in the metastatic cases [65]. A recent study indicated that ESR1 participated in the development and progression of CRC, leading to the inferior clinical outcome of CRC patients [66]. Hence, ESR1 was regarded to be an important therapeutic target against CRC. Heat shock proteins (HSPs) are commonly over-expressed in many kinds of tumors and are highly associated with a poor prognosis and therapy resistance [67]. HSP90 is one of the HSPs proteins, which has been found to restrain cell apoptosis through folding, stabilizing, and activating oncogenic proteins [68]. HSP90AA1, a member of the HSP90 family that is expressed extracellularly, is strongly associated with cancer cell invasion [69]. Therefore, targeting HSP90AA1 can be considered a good remedy for CRC therapy. Based on the predicted therapeutic targets and active compounds of AGS, we performed a molecular docking analysis for 6 active components and 5 collective targets to provide a rational explanation for the anti-CRC effect of AGS, and the results showed that AGS had a strong docking capability with SRC and ESR1. Furthermore, the results of molecular dynamic simulation also showed that these complexes fluctuate little during the simulation process, and the MD trajectories analysis, including RMSD, RMSF, and hydrogen bonding, showed that the compounds of AGS enhanced the stability of target proteins.

GO analysis showed that the numbers of BP, CC, and MF of AGS against CRC were 1079, 44, and 132, respectively. It was concluded that AGS mainly participated in the biological process of steroid metabolic process. The major reaction site was the cytoplasm in CRC treatment, and the central molecular function mainly included steroid hormone receptor activity, nuclear receptor activity, transcription factor activity, steroid binding, and endopeptidase activity. KEGG pathway analysis showed that 96 pathways in all were included and the top 10 pathways were MAPK signaling pathway, lipid and atherosclerosis, proteoglycans in cancer, prostate cancer, adherens junction, endocrine resistance, progesterone-mediated oocyte maturation, relaxin signaling pathway, FoxO signaling pathway, apoptosis, among which the MAPK signaling pathway, lipid and atherosclerosis, proteoglycans in cancer, prostate cancer, FoxO signaling pathway, and apoptosis were commonly recognized to be cancer-related. It is reported that the MAPK signaling pathway plays an important role in cell proliferation, and is commonly activated by its upstream growth-factor receptors, such as the epidermal growth factor which is commonly over-expressed in colorectal cancer [70]. The altered metabolism of lipids is a hallmark in many cancers. A series of lipid molecules, such as fatty acids, polar lipids, and oxylipins, can promote the development of CRC, and the lipid metabolism pathways have become the targets of CRC treatment [71]. Proteoglycans are a group of molecules that have a glycosaminoglycan chain. Previous studies showed that some proteoglycans, such as glypicans, agrin, and versican, play a key role in the development of liver cancer, and heparan sulfate proteoglycans were regarded to be the critical targets for the diagnosis and therapy of CRC [72, 73].

AGS has a wide range of biological properties including anti-cancer activity. Gu found that the biotransformation product S1 from AGS had significant inhibition on 6 kinds of tumor cell lines and the potential mechanism may be related to cell cycle arrest [74]. Cyclin D1, frequently over-expressed in ESR1-mutated breast cancer [75], could activate the CDK4 and CDK6 to facilitate cell cycle progression through the G1 restriction point [76, 77]. Similarly, v-SRC (viral-SRC, a transforming protein of SRC family) was found to suppress the expression of the cyclin-dependent kinase (CDK) inhibitor p27, leading to rapid transit of the G1 phase and the expressions of CDK2, CDK4, and CDK6 [78–80]. He found that the components of AGS suppressed the growth of HepG2 cells by regulating the phosphorylation of ERK, JNK, and p38 in the MAPK signaling pathway [81]. The results were consistent with our findings that MAPK1 and MAPK8 were the core proteins in AGS against CRC and the MAPK signaling pathway was the one of top 10 KEGG pathways.

In all, our study firstly investigates the anti-CRC of AGS, explores the potential mechanism, analyzes the docking patterns and binding capabilities between the active compounds and target proteins, as well as studies the stability of the complexes. The results suggest the potential application of AGS in colorectal cancer treatment or prevention for humans through integrating experimental evaluation and network study. The 11 potential active ingredients were showing a summary of the composition from AGS based on the existing database. Although the PEAGS showed higher cytotoxicity, the NBAGS and EAAGS also had good inhibitory potency against CRC cell growth. It was suspected that there probably were some unknown compounds with good anti-tumor activity, including the new compounds that have never been reported or excluded in the databases. Hence, it is needed to conduct a series of experiments to identify the constituent structures in PEAGS, which is our follow-up study.

## Conclusion

This study showed that all the three kinds of fractions from AGS, including the n-butanol extract (NBAGS), ethyl acetate fraction (EAAGS), and petroleum ether fraction (PEAGS), could significantly inhibit the proliferation of CRC cells, with the IC_50_ values of 197.24, 264.85, 15.45 µg/mL on HCT-116 cells, and 523.6, 323.59, 150.31 µg/mL on SW620 cells. Network pharmacological analysis suggested that eleven active ingredients were identified, including 4-O-galloylbergenin, 11-O-galloylbergenin, 11-O-protocatechuoylbergenin, 11-O-syringylbergenin, ardisiacrispin B, bergenin, epicatechin-3-gallate, gallic acid, quercetin, stigmasterol, stigmasterol-3-o-β-D-glucopyranoside. The PPI network showed that SRC, MAPK1, ESR1, HSP90AA1, and MAPK8 would probably be the core targets of AGS against CRC. GO analysis showed that the numbers of biological process, cellular component, and molecular function of AGS against CRC were 1079, 44, and 132, respectively, and KEGG enrichment suggested that 96 signaling pathways in all would probably be involved in AGS against CRC, among which MAPK signaling pathway, lipid and atherosclerosis, proteoglycans in cancer, prostate cancer, adherens junction would probably be the major pathways. The molecular docking study showed that the targets proteins SRC and ESR1 had strong docking capability with AGS than the other targets. The compounds of 11-O-galloylbergenin, 11-O-protocatechuoylbergenin, 11-O-syringylbergenin, epicatechin-3-gallate have higher docking scores than the ligand-protein of SRC, and the compounds of bergenin, epicatechin-3-gallate dock better than the ligand-protein of ESR1, suggesting that AGS has multiple ingredients, multiple targets, and multiple pathways against CRC. Our study for the first time investigates the anti-CRC potency of AGS, as well as uncovers the underlying mechanism. The results can probably provide valuable information for further study on the anti-CRC effect of AGS as well as its underlying mechanism.

## Supplementary Information

**Additional file 1. **Targetsof the compounds from Ardisia gigantifolia Stapf. against colorectal cancerbased on network searching.

**Additional file 2. **Thetherapeutic targets related to colorectal cancer obtained by searching theGenecards database.

**Additional file 3. **Theoverlapped targets of Ardisia gigantifolia Stapf. and colorectal cancer.

**Additional file 4. **GO Analysis.

**Additional file 5. **KEGG pathway enrichment analysis.

## Data Availability

The original contributions presented in the study are included in the article/Supplementary Material. Further inquiries can be directed to the corresponding authors.

## Abbreviations

AGS: *Ardisia Gigantifolia* Stapf.
CRC: Colorectal cancer
IARC: International agency for research on cancer
NBAGS: n-butanol fraction of AGS
EAAGS: Ethyl acetate fraction of AGS
PEAGS: Petroleum ether fraction of AGS
HNPCC: Hereditary nonpolyposis colorectal cancer
FAP: Familial adenomatous polyposis
EGFR: Epidermal growth factor receptor
VEGF: Vascular endothelial growth factor
5-FU: 5-fluorouracil
TCM: Traditional Chinese herbal medicine
GO: Gene ontology
KEGG: Kyoto encyclopedia of genes and genomes
BP: Biological process
CC: Cellular component
MF: Molecular function
ANOVA: One-way analysis of variance
PPI: Protein-protein interaction
